# First-in-human trial of an anti-5T4 antibody-monomethylauristatin conjugate, PF-06263507, in patients with advanced solid tumors

**DOI:** 10.1007/s10637-016-0419-7

**Published:** 2017-01-09

**Authors:** Geoffrey I. Shapiro, Ulka N. Vaishampayan, Patricia LoRusso, Jeremy Barton, Steven Hua, Steven D. Reich, Ronald Shazer, Carrie T. Taylor, Dawei Xuan, Hossein Borghaei

**Affiliations:** 1 Early Drug Development Center, Department of Medical Oncology, Dana-Farber Cancer Institute, 450 Brookline Ave, Mayer 446, Boston, MA 02215 USA; 2 Karmanos Cancer Institute, Detroit, MI USA; 3 Yale University, New Haven, CT USA; 4 Pfizer Oncology, La Jolla, CA USA; 5 Pfizer Early Oncology Development and Clinical Research, 10777 Science Center Drive, CB-1, San Diego, CA 92121 USA; 6 Fox Chase Cancer Center, Philadelphia, PA USA

**Keywords:** 5T4, PF-06263507, Solid tumors, Monomethylauristatin conjugate, Immunoconjugate, Antibody-drug conjugate

## Abstract

*Background* The antibody-drug conjugate PF-06263507 targets the cell-surface, tumor-associated antigen 5T4 and consists of a humanized IgG1 conjugated to the microtubule-disrupting agent monomethylauristatin-F by a non-cleavable maleimidocaproyl linker. In this first-in-human, dose-finding trial (NCT01891669), we evaluated safety, pharmacokinetics, and preliminary antitumor activity of PF-06263507 in pretreated patients with advanced solid tumors, unselected for 5T4 expression. starting at 0.05 mg/kg, with 25, 56, and 95% dose increments, depending on observed dose-limiting toxicities (DLTs), applying a modified continual reassessment method. *Results* Twenty-six patients received PF-06263507 at 0.05 to 6.5 mg/kg. The first DLT, grade 3 photophobia, occurred at 4.34 mg/kg and two additional DLTs, grade 2 keratitis and grade 1 limbal stem cell deficiency (> 2-week dosing delay), at 6.5 mg/kg. The most common adverse events (AEs) were fatigue (38.5%), photophobia (26.9%), and decreased appetite, dry eye, nausea, and thrombocytopenia (23.1% each). No treatment-related grade 4–5 AEs were reported. Systemic exposure of PF-06263507 increased in a dose-related manner. At the maximum tolerated dose (MTD, 4.34 mg/kg), mean terminal half-life for PF-06263507 and unconjugated payload were ~6 and 3 days, respectively. Payload serum concentrations were substantially lower compared with PF-06263507. No objective responses were observed. *Conclusions* The MTD and recommended phase II dose were determined to be 4.34 mg/kg. Ocular toxicities accounted for the DLTs observed, as previously reported with monomethylauristatin-F payloads. Further studies are warranted to investigate clinical activity of this agent in patients with 5T4-expressing tumors.

Trial registration ID: NCT01891669

## Introduction

Antibody-drug conjugates (ADCs) were developed to improve the therapeutic index of cytotoxic anti-cancer agents. ADCs consist of immunoconjugates in which a cytotoxic agent is chemically linked to an antibody that selectively binds to an internalizing tumor-associated antigen. This approach allows delivery of the cytotoxic agent to the tumor while minimizing exposure of normal tissues [1, 2].

5T4, also known as trophoblast glycoprotein, is a cell surface antigen that is rapidly internalized [3, 4]. Expression of 5T4, as defined by immunohistochemistry, has been observed in a variety of solid tumors (i.e., lung, breast, ovarian, endometrial, bladder, pancreatic, esophageal, and gastric cancers), whereas expression in normal, adult tissues was found to be limited [5–11]. 5T4 expression has been associated with advanced disease and/or worse clinical outcomes in patients with non-small-cell lung, colorectal, ovarian, or gastric cancer and pre-B acute lymphoblastic leukemia [7, 9–12].

**Fig. 1:**
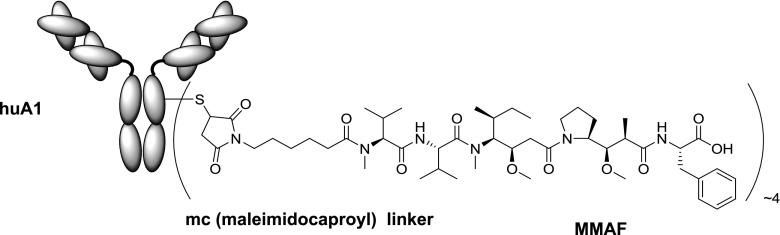
Structure of PF-06263507

PF-06263507 (5T4 ADC) is an ADC comprised of the humanized anti-5T4 IgG1 antibody PF-06281192 (huA1 mAb) conjugated via cysteine (Cys) residues to the microtubule-disrupting agent monomethylauristatin F (MMAF) by a maleimidocaproyl (mc) linker, at an average drug:antibody molecular ratio of 4:1 (Fig. 1) [2, 13–15]. PF-06281192 recognizes a conformational epitope on the extracellular domain of human and *cynomolgus* monkey 5T4. MMAF is an auristatin, a fully synthetic, pentapeptide inhibitor of tubulin polymerization that ultimately induces G2/mitosis cell cycle arrest and cell death at low picomolar intracellular concentrations. Cys-capped mc linker plus MMAF (Cys–mcMMAF, PF-06264490) constitutes the released active moiety following catabolism in the lysosome of an ADC with an mc linker to MMAF. Results from in vitro studies showed that MMAF and Cys–mcMMAF inhibited tubulin polymerization at equivalent doses, suggesting that they have comparable intracellular activity [13]. PF-06263507 was developed for the treatment of adult patients with advanced solid tumors expressing 5T4.

In vitro, the 5T4 ADC PF-06263507 and huA1 mAb PF-06281192 showed specific binding to tumor cells expressing the 5T4 antigen and rapid internalization [15]. In cell proliferation assays, PF-06263507 mediated cytotoxicity against cultured tumor cells in a 5T4-dependent manner and inhibition of tumor spheres growth in 3-dimensional culture. In preclinical studies in vivo, PF-06263507 demonstrated potent anti-tumor activity against a panel of human tumor xenografts (i.e., lung and breast cancer) with low, moderate, and high 5T4 expression levels. In contrast, treatment with the unconjugated antibody (PF-06281192) or a control ADC did not inhibit tumor growth [15].

This first-in-human, dose-finding, phase I study was designed to evaluate the safety, tolerability, pharmacokinetics (PK), and preliminary antitumor activity of PF-06263507 in patients with advanced solid malignancies.

## Methods

### Study design

This was a phase I, open-label, multi-center, single arm, dose-escalation study (NCT01891669) of single-agent PF-06263507 in sequential cohorts of adult patients with advanced solid tumors for whom no standard therapy was available; tumor 5T4 expression was not required for eligibility, based on the lack of availability of a CLIA-certified assay and because of the widespread expression of the antigen on the surface of multiple tumor types. Based on prior toxicity studies conducted in *cynomolgus* monkeys and rats (unpublished data), showing toxic effects potentially related to the Cys–mcMMAF payload on the cardiovascular system (e.g., myocardial degeneration/necrosis and/or fibrosis, premature ventricular contractions, vasculopathy), liver (e.g., multifocal sinusoidal ectasia, atrophy of hepatocytes), kidney (e.g., glomerulonephropathy and/or degeneration/regeneration of tubular epithelium), and the hematologic system (e.g., thrombocytopenia, anemia), the study protocol specified methods for assessing and monitoring potential adverse effects of PF-06263507 on these systems, including administration of initial doses in an inpatient facility to closely monitor treated patients.

The primary objective was to evaluate safety and tolerability at increasing doses of PF-06263507, determine the maximum tolerated dose (MTD) and select the recommended phase II dose (RP2D). Secondary objectives were to evaluate the overall safety profile; characterize single- and multiple-dose PK of PF-06263507, PF-06281192, and unconjugated payload (Cys–mcMMAF, PF-06264490); evaluate the immunogenicity of PF-06263507; and document any preliminary evidence of anti-tumor activity. The modified continual reassessment method (mCRM) algorithm [16, 17] was utilized to determine the MTD and run at the end of each cohort to determine whether the dose of PF-06263507 should be escalated, re-visited, or de-escalated, based on cumulative toxicity data from patients in the ongoing and all previous cohorts (see Supplemental Information for further method details).

The study protocol was approved by each participating center’s Institutional Review Board and written informed consent was obtained from each patient. The study was conducted in compliance with the Declaration of Helsinki and followed the International Congress of Harmonisation Good Clinical Practices guidelines.

### Patient entry criteria

Adult patients were included in this study if they had histological or cytological diagnosis of locally advanced or metastatic solid tumors unresponsive to treatment or with no available standard therapy; Eastern Cooperative Oncology Group (ECOG) performance status 0–1; and adequate bone marrow, renal, liver, and cardiac function. Patients were excluded if they had symptomatic or untreated brain metastases; had received major surgery, radiation treatment, or systemic anti-cancer therapy within 4 weeks prior to study entry; had previously experienced a significant allergic reaction to recombinant human or murine proteins; or had an active and clinically significant bacterial, fungal, or viral infection (i.e., hepatitis B, hepatic C, or immunodeficiency virus infection).

### Treatment and DLT

PF-06263507 was administered on day 1 of each 21-day cycle as an intravenous infusion over approximately 60 min, on an inpatient basis. Patients received PF-06263507 until disease progression, unacceptable toxicity, withdrawal of consent, or study termination. No premedication was required. Dose interruption during dosing, dose modification in the following cycle, or discontinuation were allowed by protocol in patients experiencing DLT. Intra-patient dose escalation was not permitted.

During dose escalation any of the following AEs occurring in the first 21-day treatment period and attributable to PF-06263507 were classified as DLT: a) hematologic: grade 4 neutropenia lasting > 7 days; febrile neutropenia (grade 3 neutropenia and a single body temperature > 38.3 °C or a sustained temperature of 38 °C for > 1 h); grade ≥ 3 neutropenia with infection; any-grade thrombocytopenia associated with clinically significant or life-threatening bleeding; grade 4 thrombocytopenia; b) non-hematologic: maximally treated grade ≥ 3 AEs; a confirmed positive cardiac troponin I result (> 99th percentile); delay by > 2 weeks in receiving the next scheduled treatment cycle due to persisting toxicities attributable to PF-06263507. In addition, clinically important or persisting grade 2 AEs could be considered a DLT by the investigators and the study sponsor. Grade ≥ 3 cytokine release syndrome, infusion reaction, and allergic reaction were not to be considered DLTs, but could be a reason for patient discontinuation from the study.

### Safety and efficacy assessments

Baseline evaluations, including complete blood counts, serum chemistries, vital signs, and 12-lead electrocardiograms, were performed within 4 weeks prior to the start of treatment. A follow-up visit 28–35 days after treatment discontinuation was required to monitor for any AEs. AEs were graded using the NCI CTCAE version 4.03 [18]. Computed tomography (CT) or magnetic resonance imaging (MRI) scans were obtained within 4 weeks prior to start of treatment and every 6 weeks until disease progression or end of treatment. Efficacy was assessed by Response Evaluation Criteria in Solid Tumors (RECIST) 1.1 [19].

### PK and immunogenicity assessments

Serial blood samples for PK analysis of PF-06263507, PF-06281192, and PF-06264490 were collected at multiple time points during cycles 1 and 4, pre-dose for all other cycles, and at the end of treatment. Serum concentrations of PF-06263507 and PF-06281192 were measured using validated enzyme-linked immunosorbent assays. Serum concentrations of unconjugated payload were measured with a validated liquid chromatography and tandem mass spectrometry assay. The limits of quantitation for PF-06263507, PF-06281192, and PF-06264490 were 20, 35, and 0.05 ng/mL, respectively. PK parameters for PF-06263507, PF-06281192, and PF-06264490 following intravenous infusion of PF-06263507 were calculated by non-compartmental analysis using an internally validated electronic non-compartmental analysis software (eNCA) version 2.2.4. Samples below the lower limit of quantification were set to 0 for analysis.

Immunogenicity of PF-06263507 was assessed using validated electrochemiluminescence assays, with blood samples collected pre-dose on days 1 and 15 of cycle 1, pre-dose in every cycle thereafter, and at the end of treatment. Samples positive for anti-PF-06263507 (anti-drug) antibodies (ADA) were also analyzed for neutralizing antibodies.

## Results

### Patient characteristics

Twenty-seven patients were enrolled; 1 patient died of disease progression prior to start of study treatment. Twenty-six patients received treatment with PF-06263507 at doses ranging from 0.05 to 6.5 mg/kg, between 21 August 2013 and 11 March 2015. Patients (58% women) were 25–88 years old and had been previously treated with a median of 6 (range 1–13) prior regimens. There were no meaningful differences among the treatment dose cohorts in the reported demographic or baseline patient characteristics (Table 1). Primary cancer diagnoses included colorectal cancer (*n* = 5), ovarian cancer (*n* = 4), lung cancer (*n* = 3), and cholangiocarcinoma, hepatocellular carcinoma, and pancreatic cancer (*n* = 2 each). Eight patients had different tumor types, as listed in Table 1.

**Table 1:** Patient demographics and baseline characteristics

	PF-06263507 (mg/kg)									
	0.05 *n* = 2	0.10 *n* = 2	0.19 *n* = 2	0.37 *n* = 2	0.73 *n* = 2	1.42 *n* = 2	2.78 *n* = 2	4.34 *n* = 6	5.42 *n* = 3	6.5 *n* = 3
Gender, n										
Male	1	1	1	2	1	0	0	2	1	2
Female	1	1	1	0	1	2	2	4	2	1
Age (years)										
Mean	60.5	57.0	41.0	60.0	53.5	62.0	61.5	65.2	57.7	67.7
Range	59–62	54–60	25–57	55–65	43–64	36–88	56–67	58–85	52–62	61–72
Race, n %										
White	2 (100)	1 (50)	2 (100)	2 (100)	2 (100)	2 (100)	2 (100)	6 (100)	3 (100)	2 (67)
Black	0	1 (50)	0	0	0	0	0	0	0	1 (33)
Ethnicity, n %										
Hispanic/Latino	0	0	0	0	0	0	0	0	0	1 (33)
Not Hispanic/Latino	2 (100)	2 (100)	2 (100)	2 (100)	2 (100)	2 (100)	2 (100)	6 (100)	3 (100)	2 (67)
ECOG performance status, n %										
0	0	0	0	2 (100)	0	0	0	1 (17)	0	0
1	2 (100)	2 (100)	2 (100)	0	2 (100)	2 (100)	2 (100)	5 (83)	3 (100)	3 (100)
Cancer diagnosis, n %										
Colorectal cancer	1 (50)	1 (50)	0	1 (50)	1 (50)	0	1 (50)	0	0	0
Ovarian cancer	0	0	0	0	0	1 (50)	0	1 (17)	1 (33)	1 (33)
Lung cancer	0	0	1 (50)	1 (50)	0	0	0	1 (17)	0	0
Cholangiocarcinoma	0	0	0	0	0	0	0	1 (17)	0	1 (33)
Hepatocellular carcinoma	0	0	1 (50)	0	0	0	0	1 (17)	0	0
Pancreatic cancer	0	0	0	0	1 (50)	0	0	0	0	1 (33)
Other*	1 (50) ^a^	1 (50) ^b^	0	0	0	1 (50) ^c^	1 (50) ^d^	2 (33) ^ef^	2 (67) ^gh^	0

*ECOG* Eastern Cooperative Oncology Group

*Other cancers included 1 patient each with ^a^renal cell carcinoma, ^b^cervical cancer, ^c^bladder cancer, ^d^esophageal cancer, ^e^adenoid cystic carcinoma, ^f^mesothelioma, ^g^adenoma of unknown primary, and ^h^breast cancer

### Dose assessment and DLT

Two patients each were enrolled at the PF-06263507 starting dose of 0.05 mg/kg and at increasing doses (0.10, 0.19, 0.37, 0.73, 1.42 mg/kg) up to 2.78 mg/kg with no DLTs observed. A DLT of grade 3 photophobia occurred on day 15 of cycle 1 at the 4.34 mg/kg dose level. This cohort was expanded to 6 patients and no additional DLTs were observed. Three patients treated at the 5.42 mg/kg dose level did not experience DLT. Of the 3 patients enrolled in the subsequent 6.5 mg/kg dose cohort, 1 patient had a DLT of grade 2 keratitis on day 9 of cycle 1 and 1 patient had grade 1 limbal stem cell deficiency associated with blurred vision and photophobia, at the first visit after cycle 1. As the latter patient was not retreated with PF-06263507, this constituted a treatment delay of > 2 weeks and met the definition of DLT.

The investigators and the study sponsor determined that at the 6.5 mg/kg dose level the MTD had been exceeded. One DLT at the 6.5 mg/kg dose would have led to dose de-escalation to 5.42 mg/kg and 2 DLTs in 3 patients at 6.5 mg/kg to dose de-escalation to 4.34 mg/kg, as per study design based on the mCRM algorithm (see also Supplemental Information). The MTD was determined to be 4.34 mg/kg based on the DLTs observed, the number of patients (*n* = 6) treated at this dose, and other AEs noted throughout dose escalation. At the estimated MTD, DLT occurred in 1 of 6 patients (17%).

### Safety profile

Of the 26 patients evaluable for safety, all experienced ≥ 1 treatment-emergent AE and 22 (84.6%) had ≥ 1 treatment-related AEs. The most frequently reported all causality AEs were fatigue (46.2%), decreased appetite (38.5%), nausea (30.8%), vomiting (30.8%), photophobia (26.9%), abdominal distension (23.1%), increased aspartate aminotransferase (23.1%), dry eye (23.1%), thrombocytopenia (23.1%), cough (19.2%), dyspnea (19.2%), peripheral edema (19.2%), and blurred vision (19.2%).

The most frequently observed all-grade, treatment-related AEs were fatigue (38.5%), photophobia (26.9%), decreased appetite (23.1%), dry eye (23.1%), nausea (23.1%), thrombocytopenia (23.1%), and vomiting (19.2%) (Table 2). At the MTD (*n* = 6), fatigue (*n* = 4), nausea (*n* = 3), and photophobia (*n* = 3) were the treatment-related AEs observed in more than 2 patients.

**Table 2:** Treatment-related adverse events reported in 2 or more patients

AE	Grade 1 n (%)	Grade 2 n (%)	Grade 3 n (%)	Grade 4 n (%)	Total n (%)
Any AE	15 (57.7)	4 (15.4)	3 (11.5)	0	22 (84.6)
Fatigue	9 (34.6)	1 (3.8)	0	0	10 (38.5)
Photophobia	5 (19.2)	1 (3.8)	1 (3.8)	0	7 (26.9)
Decreased appetite	5 (19.2)	1 (3.8)	0	0	6 (23.1)
Dry eye	4 (15.4)	2 (7.7)	0	0	6 (23.1)
Nausea	4 (15.4)	2 (7.7)	0	0	6 (23.1)
Thrombocytopenia	2 (7.7)	3 (11.5)	1 (3.8)	0	6 (23.1)
Vomiting	3 (11.5)	2 (7.7)	0	0	5 (19.2)
Eye pain	3 (11.5)	0	1 (3.8)	0	4 (15.4)
Increased AST	0	2 (7.7)	1 (3.8)	0	3 (11.5)
Headache	3 (11.5)	0	0	0	3 (11.5)
Blurred vision	1 (3.8)	2 (7.7)	0	0	3 (11.5)
Increased ALT	2 (7.7)	0	0	0	2 (7.7)
Anemia	1 (3.8)	1 (3.8)	0	0	2 (7.7)
Conjunctivitis	0	2 (7.7)	0	0	2 (7.7)
Dysgeusia	1 (3.8)	1 (3.8)	0	0	2 (7.7)
Increased lacrimation	2 (7.7)	0	0	0	2 (7.7)
Peripheral sensory neuropathy	2 (7.7)	0	0	0	2 (7.7)
Vitreous floaters	2 (7.7)	0	0	0	2 (7.7)

*AE* adverse event, *ALT* alanine aminotransferase, *AST* aspartate aminotransferase

All causality and treatment-related grade 3–4 AEs reported are summarized in Table 3 by dose group. Treatment-related grade 3 AEs occurred in 3 patients at the higher dose levels, including: eye pain, photophobia, and inflammation (*n* = 1; 4.34 mg/kg); increased alanine aminotransferase and increased blood alkaline phosphatase (*n* = 1; 5.42 mg/kg); and thrombocytopenia (*n* = 1, 6.5 mg/kg). One patient in the 2.78 mg/kg group had a grade 4 AE of hypercalcemia, which was not considered treatment-related. One non-treatment related death due to exacerbation of chronic obstructive pulmonary disease occurred in a patient in the 4.34 mg/kg group within 28 days of last treatment dose. No grade 4–5 treatment-related AEs were observed in patients receiving PF-06263507.

**Table 3:** All causality and treatment-related grade 3–4 adverse events

AE n, %	PF-06263507 mg/kg									
	0.1 *n* = 2		2.78 *n* = 2		4.34 *n* = 6		5.42 *n* = 3		6.5 *n* = 3	
	All	Related	All	Related	All	Related	All	Related	All	Related
Any AEs	1 (50)	0	1 (50)	0	3 (50)	1 (16.7)	1 (33.3)	1 (33.3)	2 (66.7)	1 (33.3)
Thrombocytopenia	0	0	0	0	0	0	0	0	1 (33.3)	1 (33.3)
Eye pain	0	0	0	0	1 (16.7)	1 (16.7)	0	0	0	0
Photophobia	0	0	0	0	1 (16.7)	1 (16.7)	0	0	0	0
Inflammation	0	0	0	0	1 (16.7)	1 (16.7)	0	0	0	0
Hepatobiliary disease	0	0	0	0	0	0	0	0	1 (33.3)	0
Device-related infection	0	0	0	0	1 (16.7)	0	0	0	0	0
Increased AST	0	0	0	0	0	0	1 (33.3)	1 (33.3)	0	0
Increased blood alkaline phosphatase	0	0	0	0	0	0	1 (33.3)	1 (33.3)	0	0
Hypercalcemia	0	0	1 (50)	0	0	0	0	0	0	0
Hyponatremia	1 (50.0)	0	0	0	0	0	0	0	0	0
Hypophosphatemia	0	0	0	0	1 (16.7)	0	0	0	0	0
Embolism	0	0	0	0	1 (16.7)	0	0	0	0	0

*AE* adverse event, *AST* aspartate aminotransferase; related, treatment-related

Although thrombocytopenia had been observed in the animal toxicity studies, only 1 patient (6.5 mg/kg group) developed grade 3 thrombocytopenia, on day 8 of cycle 1, which represented the nadir for this patient. Four patients had grade 2 thrombocytopenia including 2 patients each at 4.34 and 6.5 mg/kg. The nadir appeared to occur on day 8 of cycle 1, with recovery above 75 × 10^9^/L by day 15 of cycle 1.

In addition to the patient who developed an inflammatory syndrome on day 9 and grade 3 photophobia and eye pain on day 15, 10 (38.5%) patients had grade 1–2 treatment-related ocular AEs. All patients had undergone eye examinations as part of the general physical examination prior to the first dose of study drug, and no clinically significant abnormalities were found. Grade 1–2 treatment-emergent AEs occurring in > 1 patient were photophobia (23.1%); dry eye, or dry eye syndrome (23.1%); eye pain (11.5%); blurred vision (11.5%); and conjunctivitis, increased lacrimation, and vitreous floaters (each 7.7%) (Table 2). These AEs were observed as early as day 1, but usually by day 15, and as late as day 52 of treatment. The patient with inflammatory syndrome and conjunctivitis was treated with erythromycin ointment and ophthalmic prednisolone acetate, with no changes in PF-06263507 administration.

Treatment-related AEs led to study-drug discontinuation in 3 patients: 1 (4.34 mg/kg) due to photophobia; a second (6.5 mg/kg) due to bilateral annular keratitis, and the third one (6.5 mg/kg) due to limbal stem cell deficiency. Both grade 3 photophobia and grade 2 keratitis resolved without sequelae.

### Anti-tumor activity

No objective responses were observed in this study. Two patients achieved stable disease (at the lowest and the highest dose of PF-06263507), 19 patients had disease progression as best response and 2 experienced symptomatic deterioration. Best response was undetermined in 3 patients because of AE, death, or withdrawal from the study.

### Pharmacokinetics

The cycle 1 PK parameters for PF-06263507 (5T4 ADC) are summarized in Table 4. PK exposures for PF-06263507 generally increased in a dose-related manner across the 0.05–6.5 mg/kg dose range. The cycle 1 PK parameters were best characterized at the 4.34 mg/kg dose level, where data were available from 6 patients. At 4.34 mg/kg, mean clearance for PF-06263507 was ~0.7 L/day, the mean volume of distribution (V_ss_) was estimated to be 5.3 L, approximately the physiologic blood volume and similar to that of human IgG antibodies, and the mean terminal half-life (t_½_) was ~6 days.

**Table 4:** First-dose pharmacokinetic parameters for PF-06263507^a^

Dose (mg/kg)	N	C_max_ (μg/mL)	AUC_inf_ (μg × d/mL)	Terminal t_1/2_ (days)	V_ss_ (L)	CL (L/day)
0.05	2	0.8 (−)	1.8 (−)	3.0 (−)	9.5 (−)	3.4
0.10	2	2.4 (−)	4.7 (−)	1.5 (−)	3.5 (−)	1.7
0.19	2	4.0 (−)	10.4 (−)	4.5 (−)	6.2 (−)	1.5
0.37	2	9.5 (−)	24.2 (−)	3.5 (−)	6.3 (−)	1.5
0.73	2	21.2 (−)	52.3 (−)	4.4 (−)	5.0 (−)	1.0
1.42	2	45.5 (−)	90.4 (−)	3.8 (−)	5.5 (−)	1.3
2.78	2	65.7 (−)	123.7 (−)	4.2 (−)	4.2 (−)	0.9
4.34	6	102.9 (22%)	418.3 (12%)	6.0 (15%)	5.3 (16%)	0.7 (22%)
5.42	3	142.0 (48%)	332.7 (19%)	5.4 (9%)	5.6 (21%)	1.0 (23%)
6.50	3	109.1 (40%)	486.2 (−)	5.5 (−)	7.4 (−)	1.1

*C*_*max*_ maximum concentration, *AUC*_*inf*_ area under the curve from 0 to infinity, *t*_*1/2*_ terminal half-life, *V*_*ss*_ volume of distribution at steady state, *CL* clearance

^a^Data are presented as geometric mean (% coefficient of variation), with the exception of t_1/2_, which is presented as arithmetic mean (% coefficient of variation). Summary statistics are not presented if fewer than three patients had reportable parameter values

**Fig. 2:**
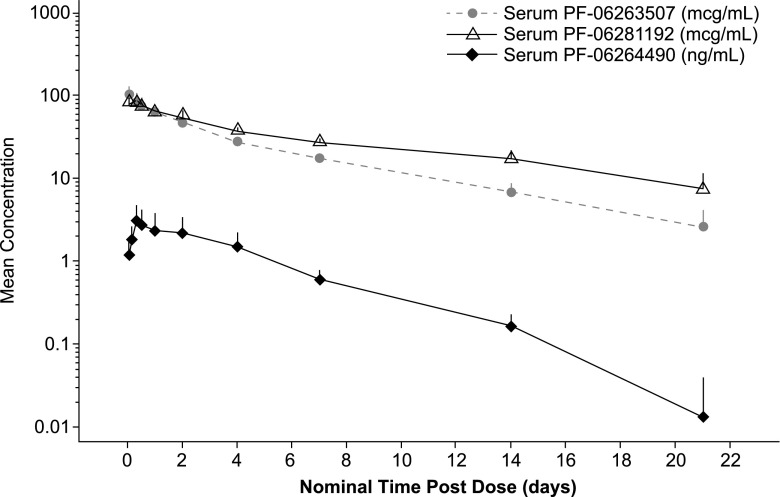
Mean serum concentration-time profiles (semi-log scale) of PF-06263507, PF-06281192, and PF-06264490 following a single 4.34 mg/kg intravenous infusion of PF-06263507 (cycle 1)

Cycle 1 mean serum concentration-time profiles for PF-06263507, PF-06281192, and PF-06264490 following a single intravenous infusion of PF-06263507 4.34 mg/kg are presented in Fig. 2. PF-06281192 (total antibody) concentration-time profiles generally resembled those of PF-06263507 at the 4.34 mg/kg dose, but with longer terminal t_½_ values of ~8.8 days. PF-06264490 (payload) serum concentrations were substantially lower compared to those observed for PF-06263507 and PF-06281192 following a 4.34 mg/kg intravenous dose of PF-06263507. PF-06264490 concentrations appeared to increase following PF-06263507 infusion and reached a mean maximum concentration (C_max_) of 3 ng/mL at ~8 h post-dose. PF-06264490 mean terminal t_½_ was 3.4 days, shorter than that observed for PF-06263507.

### Immunogenicity

Two (9%) patients tested positive for ADA at baseline (day 1, cycle 1). Of the 23 patients who were evaluated for ADA post-baseline, 4 (17%) patients were positive for ADA for at least 1 post-baseline measurement. Four (17%) patients tested positive for post-baseline neutralizing antibodies. The effect of ADA on PF-06263507 PK was not evaluated due to the small number of patients in each dose group.

## Discussion

We report here findings from the first-in-human trial of the 5T4 ADC PF-06263507 in patients with locally advanced or metastatic solid tumors with no available standard therapy following multiple lines of prior anticancer treatment.

PK exposures for PF-06263507 generally increased in a dose-related manner across the 0.05–6.5 mg/kg dose range tested. At the estimated MTD (4.34 mg/kg), the mean terminal half-life for PF-06263507 and unconjugated payload were approximately 6 and 3 days, respectively. The stability of this non-cleavable mc linker is demonstrated by the ~148-fold difference between molar AUCs of PF-06263507 and unconjugated payload observed in this study.

Patients received the first dose of PF-06263507 on an inpatient basis to allow close observation for potential toxicities. Preclinical findings of toxicity on cardiovascular, hepatic, renal, and hematologic functions (unpublished data) did not translate to clinically significant toxicities at the doses evaluated in this clinical study. PF-06263507 demonstrated a favorable safety profile with 1 DLT occurring at the estimated MTD (4.34 mg/kg), no DLTs at 5.42 mg/kg, and 2 DLTs at 6.5 mg/kg. The most frequent, treatment-related AEs at the MTD were fatigue, nausea, and photophobia. Although thrombocytopenia (mostly grade 2) was observed in some patients at the higher doses of PF-06263507 administered in this study, it did not appear to represent a significant safety concern. Furthermore, none of the patients had a > 60 msec increase from baseline in QTcF or a QTcF ≥ 500 msec, and there was no evidence of an effect of PF-06263507 on other ECG parameters (data not shown). No treatment-related grade 4–5 AEs were reported across all evaluated dose levels of PF-06263507.

Thus, overall, the non-clinical toxicity studies were not predictive of the safety profile observed for PF-06263507 in these cohorts of patients with advanced solid malignancies. However, the ocular AEs noted in this study, although not observed in previous animal safety studies conducted in *cynomolgus* monkeys and rats (unpublished data), were not unexpected, as they had been previously described in patients following administration of the CD70-targeted ADC SGN-75 and the CD19-targeted ADC SGN-CD19A. Both these ADCs comprise an MMAF payload [20–23]. As reported in 47 patients treated once every 3 weeks with SGN-75, ocular AEs (i.e., corneal epitheliopathy, dry eye) were observed in 57% of patients [20]. As in the SGN-75 and SGN-CD19A trials, patients with ocular AEs in our study were treated with artificial tears or eye drops containing steroids. Once the ocular AEs were identified, no prophylactic treatment was given to patients as the frequency and severity of these events, especially at lower doses, did not seem to warrant such therapy.

We did not observe any objective responses following treatment with PF-06263507. However, some patients may have received sub-therapeutic doses at the lower dose levels. In addition, 5T4 tumor expression was not required and not determined for the patients included in this phase I study; thus, it is possible that a number of patients had low or no 5T4 tumor expression, which could account for the lack of antitumor activity observed. A companion assay to detect 5T4 expression may, in the future, help to identify patients with the 5T4 expression levels required for PF-06263507 antitumor activity. Recent findings with the folate receptor alpha (FRα)-targeting ADC IMGN853 (mirvetuximab soravtansine) in patients with platinum-resistant epithelial ovarian cancer and with the delta-like protein 3 (DLL3)-targeted ADC SC16LD6.5 (rovalpituzumab tesirine) in patients with recurrent/refractory small-cell lung cancer indicate that expression levels of the target tumor-associated antigens may represent useful biomarkers for patient selection in ADC-based therapy [24, 25].

In addition to patient selection for tumor target expression, combination strategies are of key importance in the development of new, more effective anticancer treatment regimens and should therefore also be considered for ADC-based therapeutic approaches. PF-06263507 could represent an interesting agent for combination with different standard-of-care agents in view of its safety profile; preclinical studies have demonstrated strong synergistic or additive activity of PF-06263507 in combination with gedatolisib (PF-05212384, a pan class I isoform PI3K and mTORC1/2 inhibitor) or taxanes [15, 26–28]. Treatment with PF-06263507 plus gedatolisib resulted in induction of apoptosis and cell line-specific inhibition of the downstream biomarkers pAKT and glycogen synthase kinase. In human tumor xenografts models, dual targeting with a combination of PF-06263507 plus gedatolisib or paclitaxel produced substantially greater antitumor activity and longer survival compared with single-agent treatments, suggesting a critical role of the auristatin payload in the observed synergy. Furthermore, induction by the payloads of potentially immunogenic cell death in treated tumors [15] suggests a rationale for combining ADC-based therapy with immune checkpoint inhibitors.

The field of ADCs is rapidly expanding with currently more than 50 ADCs at different stages of clinical development. Of note, the majority of these ADCs employ a cleavable linker to attach an antibody to a tubulin-targeted payload. Cleavable linkers are associated with a “bystander effect,” whereby cleavage of the linker results in the release of a membrane-permeable, active toxin, which mediates killing of target-positive cells, but also induction of “bystander” death in neighboring, non-antigen-expressing cells. PF-06263507 was selected to comprise a 5T4 antibody linked to MMAF via a non-cleavable mc linker, as this particular construct showed an improved therapeutic potential compared with several other conjugates that used cleavable auristatins or other payloads [2, 29]. A further reduction in off-target toxicity may be achieved with antibody-drug conjugates generated by drug conjugation to genetically encoded antibody sites (site-specific conjugation), which minimizes heterogeneity, increases stability, and enhances PK properties and overall efficacy of the immunoconjugates [30].

In conclusion, the 5T4 ADC PF-06263507 was generally well tolerated at the estimated MTD of 4.34 mg/kg on a once-every-3-week schedule. At higher doses, ocular toxicities were dose-limiting. The RP2D for future studies of PF-06263507 in patients selected for 5T4 tumor expression is 4.34 mg/kg.
